# Fistules omphalo-mésenteriques; aspects épidémio logiques, diagnostiques et thérapeutiques: à propos de quatre observations au Service de Chirurgie Pédiatrique du CHU Aristide Le Dantec de Dakar

**DOI:** 10.11604/pamj.2020.37.165.19187

**Published:** 2020-10-15

**Authors:** Cheikh Seye, Pape Alassane Mbaye, Ndeye Aby Ndoye, Cheikh Diouf, Mbaye Fall, Aloïse Sagna, Oumar Ndour, Gabriel Ngom

**Affiliations:** 1 Université Alioune Diop de Bambey, Diourbel, Sénégal,; 2 Université Assane Seck de Ziguinchor, Ziguinchor 27000, Sénégal,; 3 Université Cheikh Anta Diop de Dakar, Dakar, Sénégal

**Keywords:** Fistule omphalo-mésentérique, enfant, fistulographie, Omphalomesenteric fistula, child, fistulography

## Abstract

Le but de cette étude était de déterminer les aspects épidémiologiques, diagnostiques et thérapeutiques de la fistule omphalo-mésentérique (FOM). Nous avons colligé quatre observations sur une période de 10 ans allant de janvier 2004 à décembre 2013. Les paramètres étudiés étaient la fréquence, l'âge, le sexe, les signes cliniques et radiologiques, les aspects thérapeutiques et évolutifs. La fréquence était de 0,4 cas par an. Les patients étaient âgés respectivement de 11 jours, 40 jours, 45 jours et 3 ans avec trois filles et un garçon. L´examen clinique avait retrouvé un écoulement de liquide intestinal à travers l´ombilic et un bourgeon ombilical cathétérisable dans tous les cas. Le bourgeon était prolabé chez le patient âgé de 45 jours. La fistulographie réalisée dans deux cas a permis de confirmer le diagnostic en montrant une communication entre la fistule et le grêle. Le bilan malformatif a révélé une cardiopathie congénitale cyanogène avec une communication inter ventriculaire chez l´enfant âgé de 45 jours, une malformation ano-rectale (MAR) de type cloacal associée à une fistule de l´ouraque chez le nouveau-né âgé de 11 jours. Tous les patients ont bénéficié d´un traitement chirurgical. La voie péri-ombilicale semi-circulaire était utilisée en l´absence de malformations abdomino-pelviennes associées. La communication de la fistule avec l´iléon était retrouvée dans la majorité des cas. Une résection intestinale avec anastomose termino-terminale était réalisée dans trois cas; la résection cunéiforme était faite dans un cas complet d´une exérèse complète du trajet fistuleux ouraquien et une suture vésicale, avec une colostomie de dérivation chez le nouveau-né présentant une fistule de l´ouraque et une MAR de type cloacal. Les suites opératoires étaient marquées par des crises convulsives non fébriles chez le 1^er^ enfant ayant évolué favorablement, une suppuration pariétale superficielle suivie de décès par décompensation cardiaque chez le 3^e^ cas. La fistule omphalo-mésentérique est une pathologie rare. Son diagnostic repose sur la clinique complétée par la fistulographie. Son traitement chirurgical par voie péri-ombilicale semi-circulaire donne de bons résultats. Cependant, le bilan malformatif est nécessaire.

## Abstract (en)

This study aims to determine the epidemiological, therapeutic and diagnostic features of omphalomesenteric fistulas (OMF). We conducted a study of four cases over a period of 10 years, from January 2004 to December 2013. The parameters studied were: frequency, age, sex, clinical and radiological signs, therapeutic and evolutionary features. Frequency was 0.4 cases per year. Patients were aged 11 days, 40 days, 45 days and 3 years respectively (three girls and one boy). Clinical examination showed intestinal fluid discharge from the belly button and belly button bud catheterisable in all the cases. The bud was prolapsed in the patient aged 45 days. Fistulography performed in two cases helped to confirm the diagnosis by showing a communication between the fistula and the small intestine. The assessment of malformations revealed congenital cyanogen heart disease with interventricular communication in the newborn aged 45 days, anorectal cloacal malformation associated with urachus fistula in the newborn aged 11 days. All patients underwent surgery. Semicircular periumbilical incision was performed in the absence of associated abdominopelvic malformations. A communication between the fistula and the ileum was found in the majority of cases. Bowel resection with termino-terminal anastomosis was performed in three cases. Cuneiform resection was performed in one case and was completed by complete resection of the urachal fistulous tract and bladder suture, with colostomy in newborn with urachal fistula and anorectal cloacal malformation. The postoperative course was marked by non-febrile seizures in the first child with good evolution and by superficial parietal suppuration followed by death due to cardiac decompensation in the third case. Omphalomesenteric fistula is rare. Diagnosis is based on clinical examination complemented by the fistulography. Surgical treatment using semicircular periumbilical incision gives good results. However, the assessment of malformations is necessary.

## Introduction

La fistule omphalo-mésentérique (FOM) est une anomalie d´involution totale du canal vitellin qui reste perméable sur tout son trajet [1]. C´est une malformation congénitale rare, avec une incidence qui varie entre 1/5000 et 1/15000 naissances vivantes [2]. Son diagnostic est clinique et radiologique [1]. Son traitement est chirurgical, basé sur la résection-anastomose intestinale [1]. De rares études ont été faites, avec le plus souvent un nombre de cas très limité [2]. Le but est de déterminer les aspects épidémiologiques, diagnostiques et thérapeutiques de la fistule omphalo-mésentérique au service de chirurgie pédiatrique de l´hôpital Aristide Le Dantec de Dakar.

## Étude de cas

**Observation N°1:** K.F est un nouveau-né de 11 jours, de sexe féminin adressée pour la prise en charge d´une fistule ombilicale évoluant depuis la naissance. L´examen physique notait des selles liquidiennes ombilicales avec une irritation cutanée avec un défet ombilical d´environ 1 cm laissant transparaitre un segment d´intestin grêle siège d´une petite fistule productive de selles. L´examen du périnée montrait une absence d´orifice anal, un orifice large au niveau de la vulve laissant passer des selles et des urines. Le bilan biologique montrait une anémie à 7,5g/dl, normo chrome, normocytaire. Le diagnostic d´un syndrome poly malformatif associant une fistule omphalo-mésentérique et une malformation ano-rectale type cloacal a été retenu. La cure de la fistule omphalo-mésentérique a été réalisée au premier jour d´hospitalisation. L´abord a été fait par une incision transversale sous-ombilicale. L´exploration chirurgicale a montré deux trajets fistuleux: entre l´ombilic et le jéjunum d´une part et entre l´ombilic et la vessie d´autre part. Le diagnostic per-opératoire d´une association de FOM et de fistule de l´ouraque a été retenu. Une résection cunéiforme avec suture transversale jéjunale et une exérèse complète du trajet fistuleux ouraquien avec suture vésicale ont été réalisées. Le nouveau-né a présenté au 19^e^ jour post-opératoire des crises convulsives non fébriles dues à des troubles ioniques jugulées par un traitement médical. A l´âge de 53 jours, le nourrisson a bénéficié d´une colostomie de dérivation pour la MAR cloacale avec des suites opératoires simples. La sortie de l´hôpital a été autorisée après 56 jours d´hospitalisation, avec un bilan malformatif à réaliser. L´enfant a été perdu de vue par la suite.

**Observation N°2:** M.C est un nouveau-né de 5 jours, de sexe féminin, adressée pour un écoulement de selles à travers l´ombilic. L´examen physique notait un bourgeon ombilical framboisé, centré par un orifice large d´où sort un liquide fécaloïde. Le bilan biologique a montré un taux de leucocytes de 11260 éléments/ml. L´échographie abdominale et l´échographie cardiaque n´ont pas retrouvé de malformations associées. Le diagnostic d´une fistule omphalo-mésentérique a été retenu. La cure de la fistule omphalo-mésentérique a été réalisée à l´âge de 40 jours. L´abord était fait par la voie péri-ombilicale avec une incision arciforme sous ombilicale. L´exploration chirurgicale avait retrouvé un trajet fistuleux entre l´ombilic et l´iléon. Une résection segmentaire à 2 cm de part et d´autre de la base de la fistule avec anastomose iléo-iléale a été réalisée. Les suites immédiates étaient simples. L´examen anatomo-pathologique de la pièce opératoire avait montré du tissu intestinal d´aspect normal. La sortie de l´hôpital a été autorisée au 9^e^ jour post-opératoire. L´évolution après un recul de 15 mois était sans particularité.

**Figure 1:**
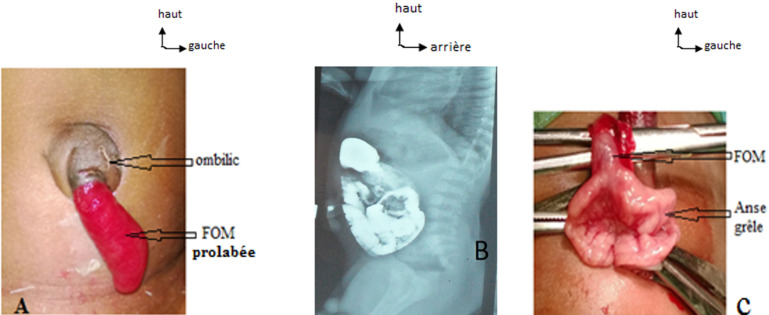
FOM prolabée à gauche (A), fistulographie au centre (B), images per-opératoires de la cure de FOM à droite (C) chez MB.D

**Figure 2:**
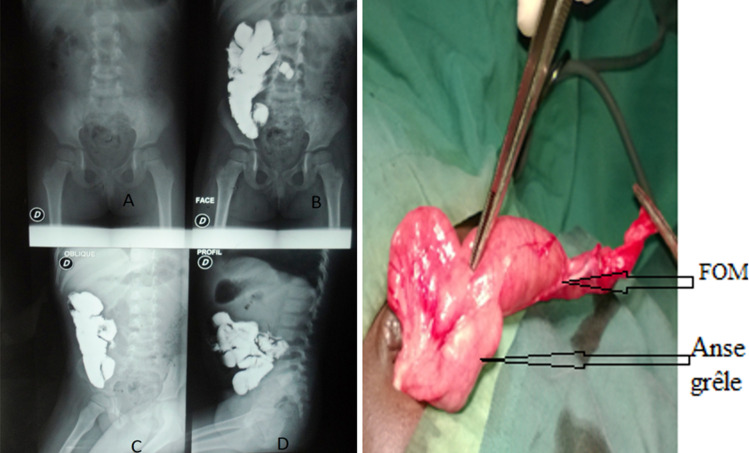
fistulographie montrant la communication entre l´ombilic et les anses à gauche et découverte peropératoire de la fistule à droite chez M.N

**Observation N°3:** MB.D est un nourrisson de 45 jours, de sexe masculin, reçu pour un écoulement de liquide et de selles à travers l´ombilic. L´examen abdominal notait un bourgeon ombilical long de 3 cm, luisant, centré par un orifice perméable laissant s´écouler un liquide fécaloïde (Figure 1). La fistulographie retrouvait une communication de l´ombilic avec les anses intestinales (Figure 1). L´échographie cardiaque couplée au doppler montrait une communication interventriculaire péri-membraneuse de 5mm avec shunt gauche-droite. Le diagnostic d´une fistule omphalo-mésentérique associé à une cardiopathie congénitale a été retenu. La cure de la fistule omphalo-mésentérique a été réalisée après une prise en charge médicale de sa cardiopathie. L´abord a été fait par la voie péri-ombilicale avec une incision arciforme sous ombilicale. L´exploration chirurgicale retrouvait le trajet fistuleux entre l´ombilic et l´iléum (Figure 2). Une résection segmentaire à deux cm de part et d´autre de la base de la fistule avec anastomose iléo-iléale a été réalisée. Nous avons décelé une suppuration pariétale de la plaie opératoire jugulée par des soins locaux. La sortie de l´hôpital était autorisée au 9^e^ jour post-opératoire accompagnée d´une référence au service de chirurgie cardio-vasculaire pour la suite de la prise en charge de sa cardiopathie congénitale. L´évolution a été marquée par le décès du patient des suites de sa cardiopathie congénitale quelques semaines après sa sortie de notre service.

**Observation N°4:** M.N est un enfant de 3 ans de sexe féminin, référée pour un écoulement de liquide à travers l´ombilic. L´examen abdominal notait un bourgeon ombilical centré par un orifice perméable laissant sourdre un liquide intestinal. Le bilan biologique était normal. La fistulographie révélait le passage du produit de contraste de l´ombilic aux anses grêles confirmant ainsi une fistule omphalo-mésentérique. L´échographie abdominale et l´écho doppler cardiaque étaient normale. Le diagnostic d´une fistule omphalo-mésentérique a été retenu et la cure a été réalisée deux mois après la première consultation. L´abord était la voie péri-ombilicale avec une incision arciforme sous ombilicale. L´exploration chirurgicale avait montré un trajet fistuleux entre l´ombilic et l´iléon. Une résection segmentaire à deux cm de part et d´autre de la base de la fistule avec anastomose iléo-iléale a été réalisée. Les suites étaient simples après un recul de 8 mois.

## Discussion

La fistule omphalo-mésentérique est une anomalie congénitale très rare [3]. Son incidence hospitalière varie dans certaines études entre 0,16 à 0,54 cas par an [1, 2, 4, 5]. Nous avons trouvé une incidence de 0,4 cas/an, ce qui correspond aux données de la littérature (Tableau 1). Dans notre étude, l´âge moyen des patients est de 10 mois avec des extrêmes de 11 jours et 3 ans. Ce résultat est semblable à ceux trouvés par certains auteurs [1, 6]. Nous n´avons pas de cas de découverte tardive au-delà de la première enfance. Dans la série de Yamada *et al*. [6], sur 65 cas, un seul cas a fait l´objet de découverte tardif à l´âge de 26 ans. La survenue de FOM est indépendante du sexe selon certains auteurs [5, 7]. Pour d´autres, il y´a une légère prédominance masculine [4, 6]. Dans notre étude, nous avons noté un sexe ratio de 0,33. Sur le plan clinique, le tableau typique de la FOM est un écoulement ombilical apparaissant dès la période néonatale, correspondant à du liquide digestif si la fistule est large, ou à des sécrétions muco-purulentes quand la fistule est plus étroite. L´examen de l´ombilic montre un bourgeon ombilical framboisé, saignant au contact, au sein duquel on peut repérer un petit orifice facilement cathétérisable [1, 6, 7]. Ce tableau était retrouvé chez tous nos patients. Parfois, la FOM peut être révélée par des complications.

**Tableau 1:** fréquence de la FOM selon divers auteurs

Auteurs	Pays	Nombre de cas	incidence
Houssain [1]	Maroc	1	0,16 cas/an
Ameh [4]	Nigéria	12	0,54 cas/an
Koylov [5]	Russie	4	0,5 cas/an
Durakbasa [2]	Turquie	5	0,31 cas/an
Notre étude	Sénégal	4	0,4 cas/an

Dans notre série, nous avons noté un cas de FOM compliquée d´un prolapsus, soit 25% des cas. Yamada *et al*. [6] rapporte 28 cas de FOM prolabée soit 52,8% des cas alors que Ameh *et al*. [4] présente un cas de prolapsus soit 8% des cas. L´intérêt de la fistulographie demeure réel pour confirmer le diagnostic mais aussi pour préciser le type de FOM [5, 8, 9]. Dans notre série, deux patients ont effectué une fistulographie permettant de confirmer la FOM et de préciser sa localisation au niveau de l´intestin grêle. Les deux autres présentaient un tableau clinique très évident de FOM. Cependant, la fistulographie reste limitée dans le diagnostic topographique entre la fistule ombilico-jéjunale et la fistule ombilico-iléale. L´association d´une FOM avec d´autres pathologies malformatives est inconstante et variable. Dans certaines séries de la littérature, aucune malformation associée n´a été décrite [2]. Par contre, dans d´autres séries, des cas isolés d´association de FOM avec d´autres malformations ont été rapportés notamment avec une omphalocèle, une fistule de l´ouraque, une polydactylie, une malrotation intestinale [8, 10-12]. Dans notre série, nous avons noté deux cas de FOM associées à d´autres malformations; l´une est associée à une malformation ano-rectale de type cloacal et une fistule de l´ouraque, l´autre à une cardiopathie congénitale cyanogène par communication interventriculaire. Le diagnostic doit être précoce pour une prise en charge adéquate afin d´éviter les complications même si une régression spontanée reste possible [9].

L´intervention chirurgicale est indispensable chaque fois que le diagnostic d´un défaut d´involution du canal omphalo-mésentérique est posé afin d´éviter les complications [1, 13]. Cependant, la voie d´abord et la méthode de résection sont variables selon le type anatomique mais aussi selon les MAP associées. Concernant la voie d´abord, la plupart des auteurs préconisent pour les FOM développées aux dépens du grêle sans MAP associées, la voie trans-ombilicale par incision péri-ombilicale circulaire à la jonction cutanéo-muqueuse avec ou sans plastie ombilicale [1, 4, 14]. En effet, elle permet de diminuer l´étendue de la plaie opératoire et elle est moins invasive. Par contre, elle ne permet pas d´explorer la cavité abdominale et surtout de préciser l´implantation exacte de la fistule sur la grêle. Récemment, pour une chirurgie moins invasive, Kozlov *et al*. [5] utilise la cœlioscopie dans le traitement des FOM chez le nouveau-né et affirme que l´âge et le poids chez l´enfant ne sont pas une contre-indication à cette méthode. Cependant, certains auteurs critiquent cette technique et pensent que les incisions effectuées pour introduire les trocarts sont aussi larges que celles réalisées pour la voie trans-ombilicale [13, 14]. Pour être encore moins invasive, et limiter l´incision sans plastie ombilicale, Giacalone *et al*. [14] préconise la voie péri-ombilicale semi-circulaire, à la partie supérieure, inférieure ou à gauche de l´ombilic. Elle permet de libérer le bourgeon ombilical après dissection circonférentielle comme dans les hernies ombilicales, de suivre la fistule jusqu´au niveau de l´intestin qui sera extériorisé. Nous avons utilisé cette dernière technique chez trois de nos quatre patients.

Les laparotomies transversale et médiane sous ombilicales et l´incision de Pfannenstiel sont indiquées en cas de cure concomitante d´une MAP associée, ou de complications [8, 11, 12]. C´est le cas chez un de nos patients qui a bénéficié d´une laparotomie transversale sous ombilicale pour une fistule de l´ouraque associée à la FOM. A l´exploration chirurgicale, nous avons noté 3 cas de FOM développées aux dépens de l´iléon (75%) et un cas aux dépens du jéjunum. Ameh *et al*. [4] dans sa série décrit 66% de fistule ombilico-iléale, 16% de fistule ombilico-colique et 8% de fistule ombilico-appendiculaire alors que Crankson *et al*. [8] rapporte un cas de fistule ombilico-appendiculaire. Cependant, l´abord ombilical reste limité dans la précision du diagnostic topographique entre la fistule ombilico-iléale et la fistule ombilico-jéjunale. En ce qui concerne la technique de résection, la plupart des auteurs préconisent la résection segmentaire suivie d´une anastomose termino-terminale pour éviter les risques de laisser en place une hétérotopie tissulaire, d´une suture sténosante ou d´une invagination secondaire [1, 3, 7, 11]. Cette technique a été utilisée chez trois de nos quatre patients.

Par contre, d´autres utilisent la résection cunéiforme avec suture transversale et aucune des complications citées ci-dessus n´a été notée [2, 5, 13]. Cette technique a été effectuée chez un de nos patients. C´est donc une alternative à la résection-anastomose surtout pour la cœlioscopie [4, 5, 15]. L´examen anatomo-pathologique réalisé chez deux de nos patients n´a pas révélé d´ectopie tissulaire. C´est le cas dans la série de Durakbasa *et al*. [2] et Ameh *et al*. [4]. Par contre, Yamada *et al*. [6] rapporte 10% d´ectopie tissulaire gastrique. Le traitement chirurgical permet la guérison dans la plupart des cas [1, 3]. Cependant, certaines complications peuvent être liées à la chirurgie notamment les infections pariétales, les occlusions sur bride, les péritonites par lâchage de suture alors que d´autres dépendent des malformations associées [1, 3-5]. Dans notre étude, nous avons noté une convulsion non fébrile traitée médicalement et une suppuration pariétale jugulée par des soins locaux. La mortalité est liée dans les différentes séries de la littérature aux complications [4, 6]. Ameh *et al*. [4] rapporte deux décès dont l´un par sepsis et l´autre en per-opératoire. Yamada *et al*. [6] rapporte 12% de décès sur une série de 65 cas. Dans notre série, nous avons noté un décès quelques semaines après l´intervention chez l´enfant porteur d´une cardiopathie congénitale cyanogène.

## Conclusion

La fistule omphalo-mésentérique est une pathologie rare. Son diagnostic repose sur la clinique complétée par la fistulographie. Son traitement chirurgical par voie péri-ombilicale semi-circulaire a donné de bons résultats. Cependant le bilan malformatif est nécessaire.
